# The Transposon-Encoded Protein TnpB Processes Its Own mRNA into ωRNA for Guided Nuclease Activity

**DOI:** 10.1089/crispr.2023.0015

**Published:** 2023-06-01

**Authors:** Suchita P. Nety, Han Altae-Tran, Soumya Kannan, F. Esra Demircioglu, Guilhem Faure, Seiichi Hirano, Kepler Mears, Yugang Zhang, Rhiannon K. Macrae, Feng Zhang

**Affiliations:** ^1^ Howard Hughes Medical Institute, Cambridge, Massachusetts, USA; ^2^ Broad Institute of MIT and Harvard, Cambridge, Massachusetts, USA; ^3^ McGovern Institute for Brain Research at MIT, Cambridge, Massachusetts, USA; ^4^ Department of Brain and Cognitive Science, Massachusetts Institute of Technology, Cambridge, Massachusetts, USA; ^5^ Department of Biological Engineering, Massachusetts Institute of Technology, Cambridge, Massachusetts, USA

## Abstract

TnpB is a member of the Obligate Mobile Element Guided Activity (OMEGA) RNA-guided nuclease family, is harbored in transposons, and likely functions to maintain the transposon in genomes. Previously, it was shown that TnpB cleaves double- and single-stranded DNA substrates in an RNA-guided manner, but the biogenesis of the TnpB ribonucleoprotein (RNP) complex is unknown. Using *in vitro* purified apo TnpB, we demonstrate the ability of TnpB to generate guide omegaRNA (ωRNA) from its own mRNA through 5′ processing. We also uncover a potential *cis*-regulatory mechanism whereby a region of the TnpB mRNA inhibits DNA cleavage by the RNP complex. We further expand the characterization of TnpB by examining ωRNA processing and RNA-guided nuclease activity in 59 orthologs spanning the natural diversity of the TnpB family. This work reveals a new functionality, ωRNA biogenesis, of TnpB, and characterizes additional members of this biotechnologically useful family of programmable enzymes.

## Introduction

Proteins associated with the IS200/605 family of transposable elements have been found to be RNA-guided DNA-targeting enzymes.^1,2^ These proteins, namely TnpB, IscB, and IsrB, associate with a noncoding RNA, termed omegaRNA or ωRNA. The ωRNA is often encoded in the same locus as the protein and enables TnpB to perform guided cleavage of DNA substrates. These IS200/605 transposon loci are flanked by transposon ends.^3^ The 3′ transposon end (right end or RE) demarcates the 3′ end of the ωRNA scaffold, which is followed by a guide sequence that is encoded outside of the transposon.^1,2^ IS200/605 family transposons are most often mobilized by TnpA, a transposase with a single catalytic tyrosine (Y1) that preferentially excises from and inserts into single-stranded DNA (ssDNA).^4^ TnpB is not known to be a transposase, but rather is thought to function akin to a homing endonuclease^5^ that prevents loss of the transposon upon excision.^1,2,6^

TnpB comprises a diverse family of proteins which have become associated with CRISPR arrays multiple times throughout evolution to give rise to various CRISPR-Cas12 subtypes.^7–10^ Biochemical experiments have shown that TnpB cleavage of double-stranded DNA (dsDNA) is dependent on both the complementarity of the guide region of the ωRNA to the target substrate and the presence of a target-adjacent motif (TAM) 5′ to the target sequence.^1,2^ This preference for a 5**′** TAM is analogous to the 5**′** protospacer-adjacent motif (PAM) preference of Cas12.^11^ In addition to cleaving dsDNA, TnpB exhibits target-specific TAM-independent cleavage of ssDNA, as well as collateral (off-target) cleavage of ssDNA substrates in the presence of target-containing dsDNA or ssDNA.^1,2^

TnpB is a promising candidate for development as a human genome editing tool due to its relatively compact size (350–550 amino acids) compared to most Cas nucleases (e.g., SpCas9 1368 amino acids (aa),^12^ SaCas9 1053 aa,^13^ AsCas12a 1307 aa).^11^ The compact domain structure of TnpB is similar to Cas12f, consisting of N-terminal recognition and wedge (WED) domains connected to the RuvC catalytic domain by a linker.^14–16^ This compact size enables packaging into adeno-associated viruses (AAVs), a clinically validated delivery modality.^17^ Although AAVs have a number of advantages for gene therapy, they have a limited packaging capacity of 4.7 kb, which is too low for most Cas enzymes and the necessary regulatory elements and guide RNA. However, small Cas enzymes such as UnCas12f1 (529 aa)^18,19^ and AsCas12f1 (422 aa)^20^ can be engineered as genome or base editors and packaged into a single AAV, highlighting the promise of compact programmable nucleases for systemically-administered gene editing therapies.

To further explore the biotechnological potential of TnpB, we sought to further characterize the biochemical activity of diverse TnpB enzymes, focusing on the formation and identity of the ωRNA. Given the broad diversity of the TnpB family, we sought to catalog and sample this diversity to explore the conserved features of TnpB function. Collectively, these results provide insight into the mechanism of TnpB and serve as a starting point for future endeavors to optimize TnpB for genome engineering.

## Materials and Methods

### Protein purification

Wild-type AmaTnpB (Addgene no. 176587) and RuvC-mutant E271A AmaTnpB were purified as described previously.^1^ TnpB with an N-terminal His14-MBP-TEV protease cleavage site was expressed in a pET45b(+) plasmid backbone in Rosetta 2(DE3) cells (Novagen). Cells were grown at 37°C in terrific broth (TB) medium supplemented with 100 μg/mL ampicillin and 30 μg/mL chloramphenicol overnight. One liter TB with 100 μg/mL ampicillin was inoculated with a 3 mL overnight culture, grown to an OD600 of 0.6–0.8, and subsequently induced with 0.2 mM IPTG and grown at 18°C for 24 h. Cells were harvested by centrifugation and resuspended in Buffer A (50 mM Tris pH 8, 1 M NaCl, 5% glycerol, 40 mM imidazole, and 5 mM β-mercaptoethanol) supplemented with benzonase (Sigma) and protease inhibitors (phenylmethylsulfonyl fluoride and Roche cOmplete, ethylenediaminetetraacetic acid-free) and then lysed by two passes with a high-pressure homogenizer (LM20 Microfluidizer, Microfluidics).

After clearing the lysate by centrifugation, the soluble fraction was bound to Ni-NTA agarose (Qiagen). The beads were first washed with Buffer B (50 mM Tris pH 8, 2 M NaCl, 5% glycerol, 40 mM imidazole, and 5 mM β-mercaptoethanol) and subsequently with Buffer A and Buffer C (50 mM Tris pH 8, 500 mM NaCl, 5% glycerol, 40 mM imidazole, and 5 mM β-mercaptoethanol). TnpB protein was then eluted in Buffer D (50 mM Tris pH 8, 500 mM NaCl, 5% glycerol, 300 mM imidazole, and 5 mM β-mercaptoethanol), incubated with 480 μg TEV protease, and dialyzed overnight against Buffer E (20 mM Tris pH 7.5, 500 mM NaCl, 5% glycerol, 0.5 mM TCEP). The protein was purified using a Resource S column (Cytiva) against a 0.2–2 M NaCl gradient. Peak fractions containing TnpB protein were pooled and dialyzed overnight against Buffer E. Protein was concentrated to 5 μM, aliquoted, snap-frozen, and stored at −80°C.

### *In vitro* RNA and DNA cleavage reactions

RNA substrates were prepared from PCR-generated DNA templates through *in vitro* transcription reactions with the NEB T7 HiScribe Kit and purified with the Zymo Clean & Concentrator-25 Kit. The 1221-nt target DNA substrate was generated by PCR and contains the cognate target sequence with a 5′ TAM of TCAC, which when cleaved generates ∼531-nt and 690-nt fragments. Sequences for RNA and DNA substrates are provided in Supplementary File S1. *In vitro* reactions were prepared with final concentrations of 1 μM TnpB, 1 μM RNA substrate(s), 10 nM DNA substrate, and 1 U/μL murine RNase inhibitor (NEB) in 20 mM HEPES and 5 mM MgCl_2_. After incubation at 55°C for 30 min, the reactions were either (1) treated with RNase A (Qiagen), Proteinase K (NEB) and purified with PCR purification columns (Qiagen) or (2) treated with DNase I (NEB) and Proteinase K (NEB). RNase-treated samples were visualized on 2% E-Gel EX agarose gels (Invitrogen), and DNase-treated samples were denatured and visualized on 6% TBE-Urea polyacrylamide gels (Thermo Fisher Scientific). The DNase-treated samples were sequenced using the NEBNext Multiplex Small RNA Library Prep Set for Illumina (NEB).

### Ortholog curation and selection

TnpBs for experimental characterization (Supplementary File S2) were sampled from diverse TnpBs by selecting different leaves from the phylogenetic tree described in a comprehensive survey of TnpB/Cas12 diversity.^8^ Selected sequences were prioritized on the basis of their available contig lengths to ensure that short range (≤3 kb) genomic associations of the transposon locus were captured appropriately, and whenever possible, sequences from complete genomes in the NCBI collection were prioritized due to their generally higher accuracy of assembled contigs relative to those from Joint Genome Institute and Whole Genome Shotgun assembled metagenomes. Locus RE boundaries (i.e., the boundary between the ωRNA scaffold and guide sequence) were determined by aligning the 3′ end of the locus with related locus sequences with MAFFT.^21^ From the alignments, the guide-scaffold boundary was identified as the downstream-most position in which a sharp drop in sequence conservation occurred.

For the phylogenetic analysis presented in Figure 3, representative TnpBs were selected from the comprehensive TnpB/Cas12 study^8^ that cover the main clades (Typical TnpBs, Derived TnpBs, and clades containing catalytic rearrangements of the RuvC-II (RII-r3 and 5) or RuvC-III (RIIIr-4) domain), major branches of TnpB, and some selected Cas12s (Supplementary File S3). These selected TnpB/Cas12 sequences, along with the experimentally studied TnpB sequences, were aligned using MAFFT-einsi,^21^ then trimmed using TrimAl^22^ with a gap threshold of 0.5. The LG+G4 substitution model for phylogenetic inference was selected using ModelFinder by optimizing the corrected Akaike Information Score.^23^ A phylogenetic tree was then inferred using IQTree2^24^ using the following parameters: -nstop 500 -bnni—ninit 5000—ntop 100—nbest 20, and 2000 ultrafast bootstraps^25^ and finally visualized with iTOL.^26^

Following this, all genes in each locus were searched using HMMER3^27^ against hidden Markov model profiles for Cas1, Cas2, Cas4,^1^ as well as Y1 TnpA, and IS607-like Serine Recombinases using PF01797 and PF00239, respectively, from PFAM.^28^ All CRISPRs were predicted using CRT.^29^ Association to CRISPR arrays, Y1 TnpA, and IS607-like Serine Recombinases was determined by the presence of the respective feature (as identified above) within 1 kb of the protein coding sequence in the genomic locus. The multiple sequence alignment was used to determine for each sequence if each of the three residues in the RuvC catalytic triad was intact by comparing if the residue aligned at the catalytic triad position matched the expected residue (D for RuvC-I, E for RuvC-II, and D for RuvC-III). Noncanonical RuvC residues are indicated by the abbreviations RII-r (RuvC-II rearrangement) and RIII-r (RuvC-III rearrangement).

### IVTT 5′ RACE

DNA templates were synthesized by Twist Biosciences or amplified by PCR from bacterial genomic DNA. All templates included a 5′ UTR containing a T7 promoter and ribosomal binding site for expression in the PURExpress *in vitro* transcription and translation (IVTT) kit (NEB). Reactions were prepared with 110 ng of DNA template and 1 U/μL murine RNase inhibitor (NEB) and incubated at 37°C for 2 h. RNA was extracted with TRIzol reagent (Thermo Fisher Scientific) and purified with Zymo Clean & Concentrator-96. 5′ rapid amplification of cDNA ends (RACE) was carried out by annealing 1 μM of primer annealing to the 3′ end of the ωRNA (Supplementary File S2) to 1000 ng of purified RNA at 65°C for 15 min and 25°C for 15 min. Next, 375 nM 5′ SR adaptor (NEB) was denatured and ligated to the RNA using T4 RNA Ligase 1 (NEB) for 1 h at 25°C. Reverse transcription was carried out using SuperScript IV RT (Invitrogen). cDNA was extracted by PCR purification (Qiagen) and amplified with 12 cycles of PCR using NEBNext High Fidelity 2X PCR Master Mix (NEB) with one primer annealing to the 5′ adaptor and one primer annealing to the 3′ end of the ωRNA, followed by a second round of PCR with 18 cycles with primers adding i7 and i5 Illumina adaptors and barcodes. Amplified libraries were gel extracted, quantified using Qubit Fluorometric Quantification (Thermo Fisher Scientific), and subjected to single-end sequencing on an Illumina MiSeq with the following parameters: read 1-300 cycles, index 1-8 cycles, index 2-8 cycles. Reads were trimmed of adaptors and aligned to template sequences using Geneious Prime.

ωRNA scaffolds were annotated by selecting sequences with a clear 5′ start site (i.e., where a substantial portion of reads corresponded to a single start site). These annotated scaffolds were used for secondary structure prediction and the TAM screen. Raw RNA-seq sequencing data can be accessed at the National Center for Biotechnology Information Sequence Read Archive (BioProject PRJNA954882). RNA secondary structure was visualized using Vienna RNAfold.^30,31^ RNA sequences were aligned using mafft-xinsi.^21^ RNAalifold^32^ and R2R^33^ were used to generate covariance models. For orthologs where no clear start site could be ascertained, the longest RNA species observed was used for the TAM screen.

### IVTT TAM screen

DNA templates encoding T7 promoter-driven TnpB proteins were generated by PCR from custom synthesis products or bacterial genomic DNA. DNA templates encoding T7 promoter-driven ωRNA scaffolds with a 20-nt guide sequence were also generated by PCR and used to prepare RNA with the T7 HiScribe Kit. IVTT reactions were prepared with 150 ng protein template, 5000 ng RNA, and 1 U/μL murine RNase inhibitor in the PURExpress kit (NEB). After 4 h at 37°C, 50 ng of an 8N TAM library plasmid was added to each reaction, and the reaction proceeded for an additional 20 min at 37°C. Reactions were treated with 10 μg RNase A (Qiagen) and 8 U Proteinase K (NEB) each followed by a 5 min incubation at 37°C. DNA was extracted by PCR purification, and adaptors were ligated using the NEBNext UltraII DNA Library Prep Kit for Illumina (NEB) with the NEBNext Adaptor for Illumina (NEB) as per the manufacturer's protocol. Following adaptor ligation, cleaved products were amplified using one primer specific to the TAM library backbone and one primer specific to the NEBNext adaptor with 12 cycles of PCR. After a second round of 18-cycle PCR with primers adding the i7 and i5 Illumina adaptors and barcodes, amplified libraries were gel-extracted, quantified using Qubit Fluorometric Quantification, and subjected to single-end sequencing on an Illumina MiSeq with the following parameters: read 1-80 cycles, index 1-8 cycles, index 2-8 cycles.

TAM enrichment was analyzed and visualized using a custom Python script.^1,34^ Raw TAM screen sequencing data can be accessed at the National Center for Biotechnology Information Sequence Read Archive (BioProject PRJNA954882).

## Results

### TnpB processes its own mRNA into ωRNA

Given the evolutionary relationship between TnpB and Cas12s, we hypothesized that the ability of Cas12s to process pre-crRNA into crRNA^35–40^ may have originated from analogous functions in TnpB. We therefore investigated whether an exemplar TnpB ortholog from *Alicyclobacillus macrosporangiidus* (AmaTnpB) possesses RNA processing activity to generate ωRNA.

**FIG. 1.**
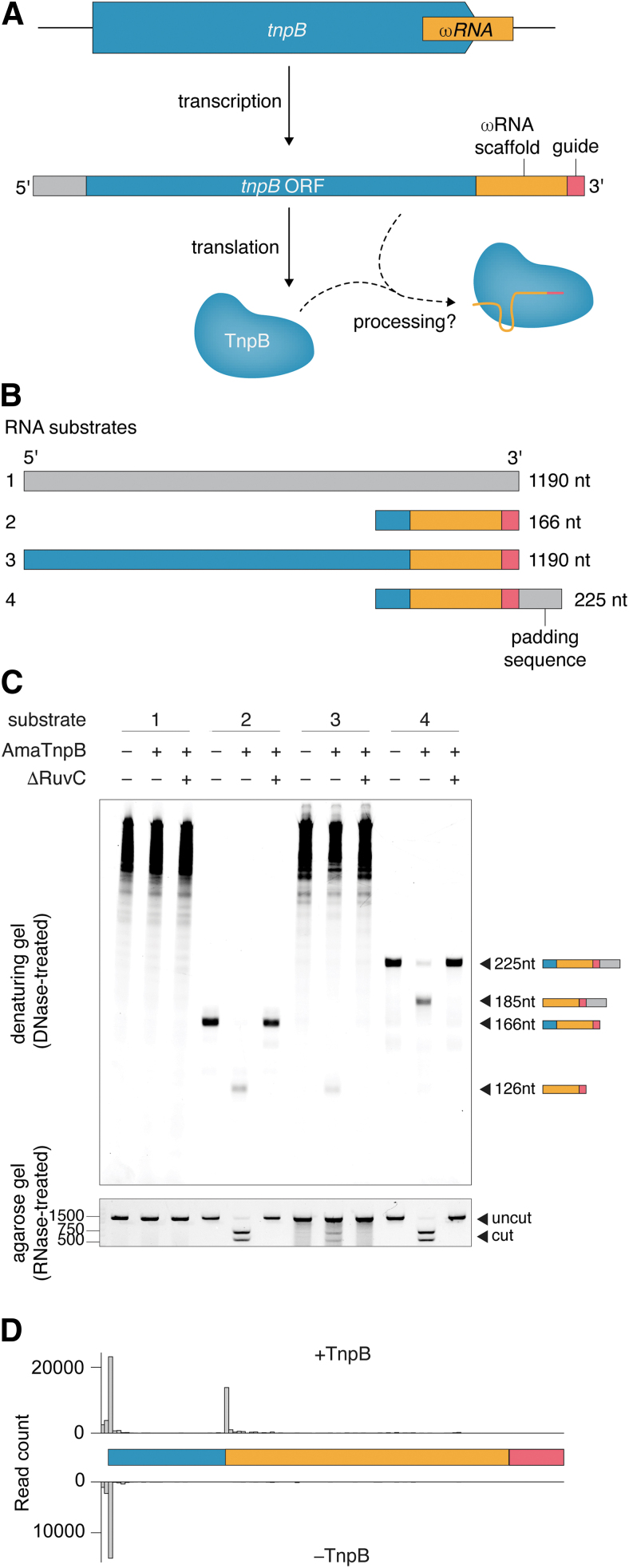
**(A)** Schematic illustrating transcription and translation of the transposon locus, whereby processing of the mRNA yields the active TnpB RNP complex. **(B)** Substrates utilized to test RNA processing activity, containing AmaTnpB coding sequence (blue), putative ωRNA scaffold (orange), guide (pink), and padding sequence (gray). **(C)** (Top) Denaturing TBE-urea gel of apo purified wild-type AmaTnpB or ΔRuvC mutant (containing a point mutant in the RuvC-II catalytic site) incubated with each of the four RNA substrates illustrated in **(B)**. Components are present in a 1:1 molar ratio of TnpB protein:ωRNA. (Bottom) Agarose E-gel demonstrating cleavage of a 1221-nt dsDNA substrate under the same conditions. Both gels are stained with SYBR gold. **(D)** RNA-seq of substrate 2 with and without active AmaTnpB *in vitro*, illustrating processing of the 5′ section from the ωRNA. Bars represent the start site of RNA species from RNA-seq. ωRNA, omegaRNA; dsDNA, double-stranded DNA; RNP, ribonucleoprotein.

To directly interrogate whether TnpB can process RNA (Fig. 1A), we incubated purified apo form wild-type AmaTnpB or a catalytically dead RuvC-II point mutant (ΔRuvC) with 1:1 molar ratios of four different *in vitro* transcribed RNA substrates, as well as a target DNA substrate (Fig. 1B). These RNA substrates include a random negative control sequence (substrate 1), the hypothesized ωRNA with a 20-nt guide sequence, which was previously determined based on RNP pulldown of a closely related ortholog^1^ (substrate 2), the full TnpB ORF extended to include the nonoverlapping ωRNA and guide sequence (substrate 3), and the hypothesized ωRNA with an additional 59 nt of 3′ padding sequence (substrate 4). After incubation, a fraction of the sample was treated with DNase to visualize the RNA species on a denaturing gel, and the remaining sample was treated with RNase to visualize cleavage of the DNA substrate.

On the denaturing gel, no processed RNA substrates were visualized from incubation of TnpB with substrate 1 (Fig. 1C). Upon TnpB incubation with RNA substrate 2, the hypothesized ωRNA sequence was processed to a 126-nt sequence, which we confirmed by RNA sequencing and hereafter refer to as the processed ωRNA (Fig. 1D). Substrate 3, which resembles the native mRNA sequence, was processed to this same 126-nt species (Fig. 1C). Substrate 4 was processed to a 185-nt species. The difference in length between the processed species resulting from substrates 2/3 and 4 suggests that the 59-nt 3′ flanking sequence is not processed by TnpB. The ΔRuvC mutant did not exhibit RNase activity on any substrate, suggesting that TnpB, and not an RNase contaminant from purification, is responsible for RNA processing and further that the RuvC domain of TnpB is responsible for ωRNA biogenesis.

We next investigated whether ωRNAs processed by TnpB support target cleavage by examining the RNase-treated fractions of the same reactions above, which include a 1221-nt dsDNA substrate containing the cognate target sequence and TAM for AmaTnpB (5′ TCAC).^1^ Wild-type TnpB utilized the processed ωRNA from substrates 2 and 3 to cleave the DNA target, and the inclusion of 3′ padding sequence in substrate 4 did not hinder substrate cleavage (Fig. 1C). These results imply that although TnpB is equipped to process 5′, but not 3′, sequence, the enzyme can perform cleavage of its DNA substrate regardless of extra sequence padding the ωRNA. This finding is consistent with the fact that TnpB from *Deinococcus radiodurans* (Dra2TnpB) utilizes only the proximal 12 nt of the guide sequence for DNA targeting, regardless of the length of guide sequence provided, due to the lack of interaction between the protein and distal end of the ωRNA: target heteroduplex.^15,16^

### *Cis*-regulation of DNA cleavage by AmaTnpB mRNA

When assaying for activity, we noticed that DNA cleavage in the presence of substrate 3 is weak compared to substrates 2 and 4 (Fig. 1C). Therefore, we explored the hypothesis that the TnpB mRNA (i.e., the extra 5′ sequence in substrate 3 compared to substrate 2) exerts an inhibitory effect on its own DNA cleavage activity.

**FIG. 2.**
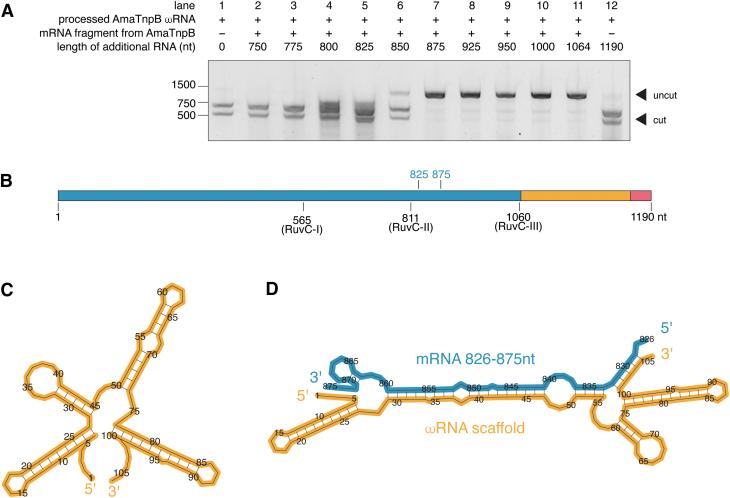
**(A)** Agarose gel of DNA cleavage by AmaTnpB with 126-nt processed ωRNA (lane 1) and additional RNA species, including various 3′ truncations of the mRNA (lanes 2–11) and a scrambled negative control (lane 12). Components are present in a 1:1:1 molar ratio of TnpB protein:mRNA:ωRNA. **(B)** Schematic of AmaTnpB mRNA, highlighting RuvC catalytic residues and hypothesized inhibitory region, which is adjacent to the RuvC-II catalytic site. **(C)** Predicted MFE secondary structure of the 106-nt processed AmaTnpB ωRNA scaffold, illustrating four stem-loop regions. **(D)** Predicted co-folding of 106-nt processed ωRNA scaffold and 50-nt inhibitory region of the AmaTnpB mRNA. MFE, minimum free energy.

We prepared reactions with active TnpB protein, the 126-nt processed ωRNA, different 3′ truncations of the mRNA, and DNA substrate (Fig. 2A). Compared to a reaction with a scrambled 1190-nt RNA (Fig. 2A lane 12) or without an additional RNA species (lane 1), the 5′ 825 nt of the mRNA does not interfere with efficient DNA cleavage. However, a sequence or structural feature present in the mRNA between 825 and 875 nt (Fig. 2B) results in a substantial reduction in DNA cleavage activity (Fig. 2A lanes 5–7). We confirmed that a 125-nt RNA fragment encompassing this region substantially reduces the DNA cleavage (Supplementary Fig. S1). We hypothesize that ωRNA secondary structure (Fig. 2C) may be disrupted by its complementarity to this region of the mRNA (Fig. 2D), pointing to a potential mechanism for the inhibitory effect.

### Extensive ωRNA diversity in survey of TnpB orthologs

We then turned our attention to the natural diversity of TnpB systems to assess the prevalence of ωRNA biogenesis and to further understand ωRNA structural and sequence diversity. TnpB is highly abundant in bacteria and archaea, and there is substantial diversity found within this family of proteins.^1,41^

**FIG. 3.**
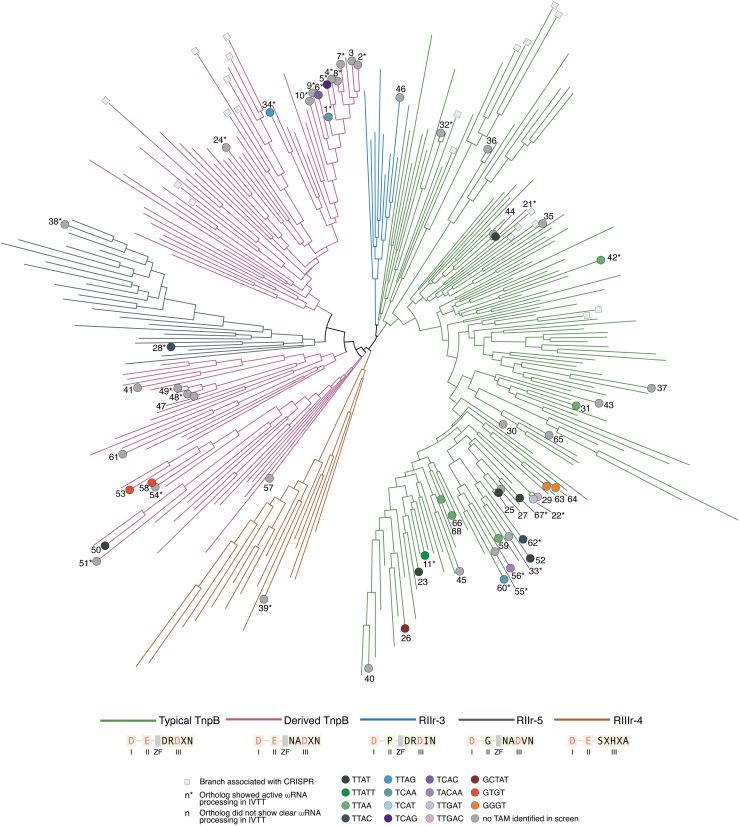
Phylogenetic tree of TnpB protein sequences from publicly available genomes and metagenomes, illustrating representative sequences from the five major clades of TnpB and CRISPR-associated TnpBs (i.e., Cas12s). A schematic of the RuvC subdomains (I, II, III, ZF) for each clade illustrates the predicted catalytic residues (pink) at each site. Fifty-nine orthologs were sampled from the tree (dots), labeled with numbers. Labels with an asterisk indicate orthologs demonstrating active ωRNA processing in IVTT reactions and whose predicted structures are illustrated in Supplementary Figure S3. Colored dots indicate 5′ TAM sequence identified from the IVTT TAM screen and are shown in more detail in Supplementary Figure S5. IVTT, *in vitro* transcription and translation; TAM, target-adjacent motif; ZF, zinc finger.

To begin experimentally studying this diversity, we subsampled the full set of TnpB systems.^8^ We focused on members of the IS200/IS605/IS607 transposon superfamily, that is, those lacking association with CRISPR arrays. We further excluded TnpBs associated with transposases besides the canonical Y1 or serine recombinases or nonmobile orthologs, as those TnpBs may perform alternate functions. We also imposed a requirement that the 3′ end of the ωRNA be well-conserved within the clade, to facilitate accurate prediction of the guide sequence. We focused on TnpBs from the five major clades, which are defined by particular configurations of the RuvC catalytic aa motif: Typical TnpBs (RuvC-III DRDXN), Derived TnpBs (RuvC-III NADXN), and clades containing catalytic rearrangements of the RuvC-II (RII-r3 and 5) or RuvC-III (RIII-r4) domain (Fig. 3).^8^ We note that there is no *a priori* expectation that TnpB proteins from these latter three clades will be catalytically inactivated; although the predicted RuvC motif is atypical, compensatory mutations may permit catalytic function, akin to the natural variation in RNase H-like domain catalytic motifs.^42^

Based on this information, we selected 59 TnpB orthologs that span the phylogenetic diversity within the constraints outlined above. These orthologs range in length from 353 to 550 aa, with some proteins having as little as 7% aa sequence identity to each other (Supplementary File S2).

To investigate these 59 TnpB orthologs at higher throughput, we expressed the TnpB-ωRNA-encoding loci from a single DNA template in IVTT reactions. As TnpB processes only the 5′ end, we utilized 5′ RACE to determine the 5′ processing site of each ωRNA. By priming cDNA synthesis from the 3′ end of the ωRNA scaffold, this technique captures the ωRNA scaffold only, excluding the guide region. We note that not all orthologs may exhibit proper expression and folding under IVTT conditions and that the absence of processing in our assay does not necessarily rule out the possibility that the ortholog has activity under different conditions.

The ωRNA sequences generated by the TnpB proteins we tested generally fall into one of two categories: those with at least one clear 5′ processing site (30/59 orthologs) and those without a clear site based on the coverage plots of the RNA reads (Supplementary Fig. S2). The IVTT 5′ RACE assay recapitulated the AmaTnpB *in vitro* processing experiments, generating a 106-nt scaffold (ortholog 6 in Supplementary Fig. S2). Some orthologs, such as Dra2TnpB (ortholog 22), showed multiple apparent processing sites, consistent with previous observations suggesting either incomplete or promiscuous RNase activity.^2,15^ The 30 orthologs with evidence of processing ability are found throughout the tree and are not confined to specific clades (Fig. 3).

**FIG. 4.**
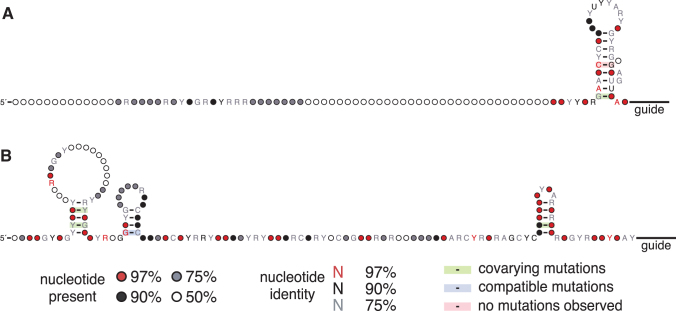
**(A)** Covariance model of ωRNA scaffold structures from 11 orthologs in the clade of Typical TnpBs. In this clade, the only conserved structure is the 3′ stem-loop within 10 nt of the start of the guide sequence. **(B)** Covariance model of ωRNA scaffold structures of 16 orthologs in the clade of Derived TnpBs. In this clade, two 5′ stem loops, as well as the 3′ stem loop, are conserved.

Processed ωRNA species ranged from 79 to 466 nt and have predicted structures rich in hairpins, of which the number and relative orientation vary widely. The RE of IS200/605 transposons is known to contain a subterminal hairpin, which we find in almost all orthologs within 10 nt of the 3′ end of the ωRNA scaffold (Fig. 4, Supplementary Fig. S3). This appears to be the only consistently conserved feature among these divergent TnpB ωRNA scaffolds. The transposase TnpA is known to interact with subterminal hairpins at the 5′ and 3′ ends of the transposon,^43^ but the 3′ hairpin may be functionally important for TnpB as well. Recently solved cryo-EM structures of Dra2TnpB illustrate how a linker in the WED domain interacts with the stem of this 3′ hairpin.^15,16^ Furthermore, this interaction is present in experimentally solved structures of all Cas12 subtypes to date, pointing to an evolutionarily conserved nuclease-guide RNA interaction (Supplementary Fig. S4).^14,15,37,39,44–48^

### Limited diversity of TAM sequences

We utilized the newly-determined ωRNA sequences to conduct a screen for DNA nuclease activity of the 59 TnpB orthologs in IVTT reactions to ascertain 5′ TAM preferences. For orthologs without a clear 5′ start site of the ωRNA, we utilized the longest RNA species observed in the sequencing. Using this screen, we recovered the known TAM sequences of TnpB orthologs characterized previously, including AmaTnpB (TCAC)^1^ and Dra2TnpB (TTGAT).^2^ In total, 27 out of 59 orthologs demonstrated TAM activity in the screen; these active orthologs are broadly distributed among the different TnpB clades and branches (Fig. 3). One ortholog in the RII-r5 clade is active, confirming that catalytic rearrangements of the RuvC catalytic site can still support guided dsDNA cleavage. We noted several orthologs that process ωRNA but do not show activity in the IVTT TAM screen and speculate that these TnpBs may target alternate substrates besides dsDNA, consistent with the diversity of substrates targeted by Cas12s.^49,50^

TAMs were found to be a maximum of 5 nt long, with relatively little degeneracy in the positions (Supplementary Fig. S5). Nearly all characterized TAMs are AT-rich, although a small subset is G-rich. This relative lack of diversity in the TAM sequence is striking compared to, for example, Cas9, which is a relatively less abundant and diverse protein family compared to TnpB^1,8^ but still exhibits a wide variation in PAMs.^51^ The limited TAM diversity of TnpBs may be explained by the co-evolution of TnpA and TnpB, as TnpA also utilizes the TAM to recognize the transposon ssDNA for excision and insertion.^1,2,6,43,52^ Although it is not yet clear how, if at all, TnpB interacts with TnpA and the transposon DNA, the fact that both proteins possess the same TAM sequence is a clear constraint on the TAM diversity.

## Discussion

The biochemical activity of OMEGA nucleases was previously found to include RNA-guided ssDNA and dsDNA cleavage, but no RNAse activity was evident.^1^ Although heterologous expression of TnpB loci in *Escherichia coli* generates processed ωRNA species that physically associate with TnpB protein, it was unclear whether these RNA species were the result of TnpB RNA processing activity or endogenous host RNases.^1,2,15^

In this study, we show that TnpB can process its own mRNA into ωRNA to generate active RNP complexes. In the cell, it is possible that endogenous host RNases assist in processing, especially at the 3′ end of the guide. We observed that an intact RuvC catalytic domain is essential for TnpB ωRNA processing activity. We therefore infer that the TnpB RuvC domain performs the nucleophilic attack on the RNA, which is consistent with the activities of Cas12c2^37^ and Cas12j^40^ but in contrast to Cas12a^35,36^ and Cas12i,^38,39^ which instead use the WED domain for crRNA processing. These findings attribute the additional function of RNA processing to this class of RNA-guided nucleases.

The observation that part of the AmaTnpB mRNA exerts an inhibitory effect on cleavage of DNA raises the question of whether this phenomenon is conserved among different TnpB orthologs. IS200/605 transposons are known to contain a variety of post-transcriptional *cis*-regulatory mechanisms to regulate TnpA activity, including mRNA secondary structure and an antisense small RNA,^53^ suggesting that TnpB activity may also be regulated by similar mechanisms. For TnpB, the inhibitory region of the mRNA may base pair with the ωRNA, thereby disrupting its structure and ability to bind to TnpB. The inhibitory effect may also be acting on the RNP complex through a different mechanism. Whether this inhibitory effect can be recapitulated in cells and its functional consequence remains to be explored.

According to recent evidence, TnpB improves retention of its transposon whereby an ωRNA expressed from one transposon locus targets transposon-lacking versions of that locus in the same cell, that is, loci where the transposon has not yet inserted or those which have undergone excision.^6^ Therefore, it appears that TnpB DNA cleavage serves to fix the transposon-containing locus in the population by eliminating loci that have undergone excision^1^ and/or homing to un-inserted loci.^2,54^ Under this hypothesis, the TnpB mRNA could serve as a temporally-sensitive signal of active transcription of the transposon and its continued presence in the genome and, therefore, exert negative feedback on TnpB DNA cleavage to prevent unnecessary genome instability.

We also note that the negative feedback exerted by TnpB mRNA on DNA cleavage should be taken into account when utilizing TnpB for applications in heterologous systems. Codon optimization of the TnpB ORF sequence up until the 5′ end of the ωRNA will likely abrogate the inhibitory effect, ultimately maximizing enzymatic activity.

Our exploration of 59 different TnpB loci reconstituted in IVTT revealed a substantial amount of diversity in the ωRNAs of these orthologs. Among these orthologs, the ωRNAs contain a minimum of two predicted stem-loop structures but otherwise vary widely in length and overall topology. Furthermore, parts of the ωRNA with predicted secondary structure may in fact be disordered or flexible, as was found with Dra2TnpB, for which a fully functional ωRNA can be reconstituted by maintaining the structured domains of the ωRNA.^15^

Overall, our demonstration of TnpB RNA processing and observation of a *cis*-regulatory mechanism complement studies of the biological function of OMEGA effectors. Furthermore, our survey of the natural diversity of TnpB-ωRNA complexes adds to the toolbox of this biotechnologically promising family of enzymes.

## Supplementary Material

Supplemental data

Supplemental data

Supplemental data

Supplemental data

## References

[1] Altae-Tran H, Kannan S, Demircioglu FE, et al. The widespread IS200/IS605 transposon family encodes diverse programmable RNA-guided endonucleases. Science 2021;374(6563):57–65; doi: 10.1126/science.abj6856 34591643 PMC8929163

[2] Karvelis T, Druteika G, Bigelyte G, et al. Transposon-associated TnpB is a programmable RNA-guided DNA endonuclease. Nature 2021;599(7886):692–696; doi: 10.1038/s41586-021-04058-1 34619744 PMC8612924

[3] Siguier P, Gourbeyre E, Chandler M. Bacterial insertion sequences: Their genomic impact and diversity. FEMS Microbiol Rev 2014;38(5):865–891; doi: 10.1111/1574-6976.12067 24499397 PMC7190074

[4] Ton-Hoang B, Guynet C, Ronning DR, et al. Transposition of ISHp608, member of an unusual family of bacterial insertion sequences. EMBO J 2005;24(18):3325–3338; doi: 10.1038/sj.emboj.7600787 16163392 PMC1224677

[5] Stoddard BL. Homing endonuclease structure and function. Q Rev Biophys 2005;38(1):49–95; doi: 10.1017/S0033583505004063 16336743

[6] Meers C, Le H, Pesari SR, et al. Transposon-encoded nucleases use guide RNAs to selfishly bias their inheritance. bioRxiv 2023;2023.03.14.532601; doi: 10.1101/2023.03.14.532601

[7] Shmakov S, Smargon A, Scott D, et al. Diversity and evolution of class 2 CRISPR-Cas systems. Nat Rev Microbiol 2017;15(3):169–182; doi: 10.1038/nrmicro.2016.184 28111461 PMC5851899

[8] Altae-Tran H, Shmakov S, Makarova KS, et al. Diversity, evolution, and classification of the RNA-guided nucleases TnpB and Cas12. n.d. 10.1073/pnas.2308224120 PMC10691335 37983496

[9] Shmakov S, Abudayyeh OO, Makarova KS, et al. Discovery and functional characterization of diverse class 2 CRISPR-Cas systems. Mol Cell 2015;60(3):385–397; doi: 10.1016/j.molcel.2015.10.008 26593719 PMC4660269

[10] Makarova KS, Wolf YI, Iranzo J, et al. Evolutionary classification of CRISPR–Cas systems: A burst of class 2 and derived variants. Nat Rev Microbiol 2019;18(2):67–83; doi: 10.1038/s41579-019-0299-x 31857715 PMC8905525

[11] Zetsche B, Gootenberg JS, Abudayyeh OO, et al. Cpf1 is a single RNA-guided endonuclease of a class 2 CRISPR-Cas system. Cell 2015;163(3):759–771; doi: 10.1016/j.cell.2015.09.038 26422227 PMC4638220

[12] Cong L, Ran FA, Cox D, et al. Multiplex genome engineering using CRISPR/Cas systems. Science 2013;339(6121):819–823; doi: 10.1126/science.1231143 23287718 PMC3795411

[13] Ran FA, Cong L, Yan WX, et al. In vivo genome editing using Staphylococcus aureus Cas9. Nature 2015;520(7546):186–191; doi: 10.1038/nature14299 25830891 PMC4393360

[14] Takeda SN, Nakagawa R, Okazaki S, et al. Structure of the miniature type V-F CRISPR-Cas effector enzyme. Mol Cell 2021;81(3):558.e3–570.e3; doi: 10.1016/j.molcel.2020.11.035 33333018

[15] Nakagawa R, Hirano H, Omura SN, et al. Cryo-EM structure of the transposon-associated TnpB enzyme. Nature 2023;616(7956):390–397; doi: 10.1038/s41586-023-05933-9 37020030 PMC10097598

[16] Sasnauskas G, Tamulaitiene G, Druteika G, et al. TnpB structure reveals minimal functional core of Cas12 nuclease family. Nature 2023;616(7956):384–389; doi: 10.1038/s41586-023-05826-x 37020015

[17] Au HKE, Isalan M, Mielcarek M. Gene therapy advances: A meta-analysis of AAV usage in clinical settings. Front Med 2021;8:809118; doi: 10.3389/fmed.2021.809118 PMC8864161 35223884

[18] Kim DY, Lee JM, Moon SB, et al. Efficient CRISPR editing with a hypercompact Cas12f1 and engineered guide RNAs delivered by adeno-associated virus. Nat Biotechnol 2021; doi: 10.1038/s41587-021-01009-z 10.1038/s41587-021-01009-z PMC8763643 34475560

[19] Kim DY, Chung Y, Lee Y, et al. Hypercompact adenine base editors based on transposase B guided by engineered RNA. Nat Chem Biol 2022;18:1005–1013; doi: 10.1038/s41589-022-01077-5 35915259

[20] Wu Z, Zhang Y, Yu H, et al. Programmed genome editing by a miniature CRISPR-Cas12f nuclease. Nat Chem Biol 2021; doi: 10.1038/s41589-021-00868-6 10.1038/s41589-021-00868-6 34475565

[21] Katoh K, Standley DM. MAFFT multiple sequence alignment software version 7: Improvements in performance and usability. Mol Biol Evol 2013;30(4):772–780; doi: 10.1093/molbev/mst010 23329690 PMC3603318

[22] Capella-Gutiérrez S, Silla-Martínez JM, Gabaldón T. trimAl: A tool for automated alignment trimming in large-scale phylogenetic analyses. Bioinformatics 2009;25(15):1972–1973; doi: 10.1093/bioinformatics/btp348 19505945 PMC2712344

[23] Kalyaanamoorthy S, Minh BQ, Wong TKF, et al. ModelFinder: Fast model selection for accurate phylogenetic estimates. Nat Methods 2017;14(6):587–589; doi: 10.1038/nmeth.4285 28481363 PMC5453245

[24] Nguyen L-T, Schmidt HA, von Haeseler A, et al. IQ-TREE: A fast and effective stochastic algorithm for estimating maximum-likelihood phylogenies. Mol Biol Evol 2015;32(1):268–274; doi: 10.1093/molbev/msu300 25371430 PMC4271533

[25] Hoang DT, Chernomor O, von Haeseler A, et al. UFBoot2: Improving the ultrafast bootstrap approximation. Mol Biol Evol 2018;35(2):518–522; doi: 10.1093/molbev/msx281 29077904 PMC5850222

[26] Letunic I, Bork P. Interactive tree of life (iTOL) v3: An online tool for the display and annotation of phylogenetic and other trees. Nucleic Acids Res 2016;44(W1):W242–5; doi: 10.1093/nar/gkw290 27095192 PMC4987883

[27] Mistry J, Finn RD, Eddy SR, et al. Challenges in homology search: HMMER3 and convergent evolution of coiled-coil regions. Nucleic Acids Res 2013;41(12):e121; doi: 10.1093/nar/gkt263 23598997 PMC3695513

[28] Mistry J, Chuguransky S, Williams L, et al. Pfam: The protein families database in 2021. Nucleic Acids Res 2021;49(D1):D412–D419; doi: 10.1093/nar/gkaa913 33125078 PMC7779014

[29] Bland C, Ramsey TL, Sabree F, et al. CRISPR recognition tool (CRT): A tool for automatic detection of clustered regularly interspaced palindromic repeats. BMC Bioinform 2007;8:209; doi: 10.1186/1471-2105-8-209 PMC1924867 17577412

[30] Gruber AR, Lorenz R, Bernhart SH, et al. The Vienna RNA websuite. Nucleic Acids Res 2008;36(Web Server issue):W70-4; doi: 10.1093/nar/gkn188 18424795 PMC2447809

[31] Wiegreffe D, Alexander D, Stadler PF, et al. RNApuzzler: Efficient outerplanar drawing of RNA-secondary structures. Bioinformatics 2019;35(8):1342–1349; doi: 10.1093/bioinformatics/bty817 30239566

[32] Bernhart SH, Hofacker IL, Will S, et al. RNAalifold: Improved consensus structure prediction for RNA alignments. BMC Bioinform 2008;9:474; doi: 10.1186/1471-2105-9-474 PMC2621365 19014431

[33] Weinberg Z, Breaker RR. R2R—software to speed the depiction of aesthetic consensus RNA secondary structures. BMC Bioinform 2011;12:3; doi: 10.1186/1471-2105-12-3 PMC3023696 21205310

[34] Tareen A, Kinney JB. Logomaker: Beautiful sequence logos in Python. Bioinformatics 2020;36(7):2272–2274; doi: 10.1093/bioinformatics/btz921 31821414 PMC7141850

[35] Fonfara I, Richter H, Bratovič M, et al. The CRISPR-associated DNA-cleaving enzyme Cpf1 also processes precursor CRISPR RNA. Nature 2016;532(7600):517–521; doi: 10.1038/nature17945 27096362

[36] Swarts DC, van der Oost J, Jinek M. Structural basis for guide RNA processing and seed-dependent DNA targeting by CRISPR-Cas12a. Mol Cell 2017;66(2):221.e4–233.e4; doi: 10.1016/j.molcel.2017.03.016 28431230 PMC6879319

[37] Kurihara N, Nakagawa R, Hirano H, et al. Structure of the type V-C CRISPR-Cas effector enzyme. Mol Cell 2022;82(10):1865.e4–1877.e4; doi: 10.1016/j.molcel.2022.03.006 35366394 PMC9522604

[38] Zhang H, Li Z, Xiao R, et al. Mechanisms for target recognition and cleavage by the Cas12i RNA-guided endonuclease. Nat Struct Mol Biol 2020;27(11):1069–1076; doi: 10.1038/s41594-020-0499-0 32895556 PMC8256696

[39] Huang X, Sun W, Cheng Z, et al. Structural basis for two metal-ion catalysis of DNA cleavage by Cas12i2. Nat Commun 2020;11(1):5241; doi: 10.1038/s41467-020-19072-6 33067443 PMC7567891

[40] Pausch P, Al-Shayeb B, Bisom-Rapp E, et al. CRISPR-CasΦ from huge phages is a hypercompact genome editor. Science 2020;369(6501):333–337; doi: 10.1126/science.abb1400 32675376 PMC8207990

[41] Kapitonov VV, Makarova KS, Koonin EV. ISC, a novel group of bacterial and archaeal DNA transposons that encode Cas9 homologs. J Bacteriol 2015;198(5):797–807; doi: 10.1128/JB.00783-15 26712934 PMC4810608

[42] Majorek KA, Dunin-Horkawicz S, Steczkiewicz K, et al. The RNase H-like superfamily: New members, comparative structural analysis and evolutionary classification. Nucleic Acids Res 2014;42(7):4160–4179; doi: 10.1093/nar/gkt1414 24464998 PMC3985635

[43] Guynet C, Hickman AB, Barabas O, et al. In vitro reconstitution of a single-stranded transposition mechanism of IS608. Mol Cell 2008;29(3):302–312; doi: 10.1016/j.molcel.2007.12.008 18280236

[44] Yamano T, Nishimasu H, Zetsche B, et al. Crystal structure of Cpf1 in complex with guide RNA and target DNA. Cell 2016;165(4):949–962; doi: 10.1016/j.cell.2016.04.003 27114038 PMC4899970

[45] Yang H, Gao P, Rajashankar KR, et al. PAM-dependent target DNA recognition and cleavage by C2c1 CRISPR-Cas endonuclease. Cell 2016;167(7):1814.e12–1828.e12; doi: 10.1016/j.cell.2016.11.053 27984729 PMC5278635

[46] Liu J-J, Orlova N, Oakes BL, et al. CasX enzymes comprise a distinct family of RNA-guided genome editors. Nature 2019;566(7743):218–223; doi: 10.1038/s41586-019-0908-x 30718774 PMC6662743

[47] Li Z, Zhang H, Xiao R, et al. Cryo-EM structure of the RNA-guided ribonuclease Cas12g. Nat Chem Biol 2021;17(4):387–393; doi: 10.1038/s41589-020-00721-2 33495647 PMC8256697

[48] Park J-U, Tsai AW-L, Rizo AN, et al. Structures of the holo CRISPR RNA-guided transposon integration complex. Nature 2023;613(7945):775–782; doi: 10.1038/s41586-022-05573-5 36442503 PMC9876797

[49] Yan WX, Hunnewell P, Alfonse LE, et al. Functionally diverse type V CRISPR-Cas systems. Science 2019;363(6422):88–91; doi: 10.1126/science.aav7271 30523077 PMC11258546

[50] Wang JY, Pausch P, Doudna JA. Structural biology of CRISPR-Cas immunity and genome editing enzymes. Nat Rev Microbiol 2022;20(11):641–656; doi: 10.1038/s41579-022-00739-4 35562427

[51] Gasiunas G, Young JK, Karvelis T, et al. A catalogue of biochemically diverse CRISPR-Cas9 orthologs. Nat Commun 2020;11(1):5512; doi: 10.1038/s41467-020-19344-1 33139742 PMC7606464

[52] Lavatine L, He S, Caumont-Sarcos A, et al. Single strand transposition at the host replication fork. Nucleic Acids Res 2016;44(16):7866–7883; doi: 10.1093/nar/gkw661 27466393 PMC5027513

[53] Ellis MJ, Trussler RS, Haniford DB. A cis-encoded sRNA, Hfq and mRNA secondary structure act independently to suppress IS200 transposition. Nucleic Acids Res 2015;43(13):6511–6527; doi: 10.1093/nar/gkv584 26044710 PMC4513863

[54] Kaur D, Kuhlman TE. IS200/IS605 family-associated TnpB increases transposon activity and retention. bioRxiv 2022;2022.10.12.511977; doi: 10.1101/2022.10.12.511977

