# Supplementary material for: The Transposon-Encoded Protein TnpB Processes Its Own mRNA into ωRNA for Guided Nuclease Activity
Source: CRISPR J. 2023 Jun 1;6(3):232–42. doi: 10.1089/crispr.2023.0015 (PMC10278001; doi:10.1089/crispr.2023.0015)
Supplement: Supplemental data [file Supp_FigureS1_S5.zip › Supplementary Figure Legends.docx]

Supplementary Material

**Figure S1**

Agarose gel of DNA cleavage by AmaTnpB with 126-nt processed ωRNA (lane 1) and additional RNA species including various truncations of the mRNA (lanes 2-4, 6-12) and a scrambled negative control (lane 5). DNA cleavage inhibition is observed when the first 875 nt of the mRNA are included in the reaction, but not the first 825 nt. Addition of different 5′ truncations of the 875-nt species reveals an inhibitory effect when nucleotides 751-875 of the mRNA are included in the reaction, or any RNA species encompassing this region. Components are present in a 1:1:1 molar ratio of TnpB protein:mRNA:ωRNA.

**Figure S2**

ωRNA sequencing of 59 orthologs showing coverage of RNA reads after transcription and translation of TnpB loci in IVTT. Each plot is labeled with the ortholog ID. Locus schematics are shown below each coverage trace with the TnpB ORF in blue and ωRNA scaffold in orange.

**Figure S3**

1. Predicted minimum free energy (MFE) ωRNA scaffold structures of 11 orthologs in the clade of Typical TnpBs. Blue circles mark the 3′ end of the scaffold, indicating where the guide sequence would begin. Orange highlights indicate stem structures at the 3′ end that likely interact with the TnpB WED domain. ωRNA sequences are arranged by phylogenetic proximity of the TnpB proteins.
2. Predicted MFE ωRNA scaffold structures of 16 orthologs in the clade of Derived TnpBs.
3. Predicted MFE ωRNA scaffold structures of 2 catalytically rearranged orthologs in the RII-r5 clade.
4. Predicted MFE ωRNA scaffold structures of 1 catalytically rearranged ortholog in the RIII-r4 clade.

**Figure S4**

1. Interactions between ωRNA/crRNA and Dra2TnpB (TnpB) or Cas12 subtypes. TnpB and Cas12 are illustrated by their common regions, the REC lobe (blue colors) and NUC lobe (green colors). Thick black or gray lines indicate interactions of ωRNA/crRNA with the protein. The black interaction highlights how a 3′ hairpin region in ωRNA or crRNA contacts the WED domain. PI/TI, PAM/TAM-interacting; REC, recognition; BH, bridge helix; NUC, nuclease; crRNA, CRISPR RNA.
2. The 3′ hairpin of the RNA (black) interacts with a conserved linker (yellow) in the WED domain.
3. Conservation of the interaction between the 3′ hairpin (black) and the WED loop (yellow) in experimentally solved Dra2TnpB[^15^](https://paperpile.com/c/XHTflN/ux2N) and 8 Cas12 structures.[^14,37,39,44–48^](https://paperpile.com/c/XHTflN/ZOyl+jwZP+z2Od+0mET+ThmL+eGUV+wvjH+8WHb)
4. Evolutionary model of TnpB/Cas12 and ωRNA/crRNA. Given that the hairpin-WED interaction is conserved in TnpB and Cas12s, it is possible that TnpB emerged from an ancestral WED-containing protein binding to a conserved RNA hairpin. This ancestral RNP could have interacted with and eventually fused with a RuvC-like protein, with diverse RNA-protein interactions arising thereafter.

**Figure S5**

Sequence logos illustrating 5′ TAM preference of 27 orthologs with nuclease activity. The -1 position represents the base directly 5′ to the target site.

**Supplementary File 1**

Sequences for RNA and DNA substrates used in gels in Figures 1, 2, and S1.

**Supplementary File 2**

Sequence information for 59 experimentally tested TnpB orthologs, including accession numbers, primers, and experimentally determined ωRNA sequences.

**Supplementary File 3**

Sequences for 294 proteins, including 59 experimentally tested TnpB orthologs and 236 TnpB and Cas12 representatives, that comprise the phylogenetic tree in Figure 3.
