# Supplementary figures and images for: The Transposon-Encoded Protein TnpB Processes Its Own mRNA into ωRNA for Guided Nuclease Activity
Source: CRISPR J. 2023 Jun 1;6(3):232–42. doi: 10.1089/crispr.2023.0015 (PMC10278001; doi:10.1089/crispr.2023.0015)

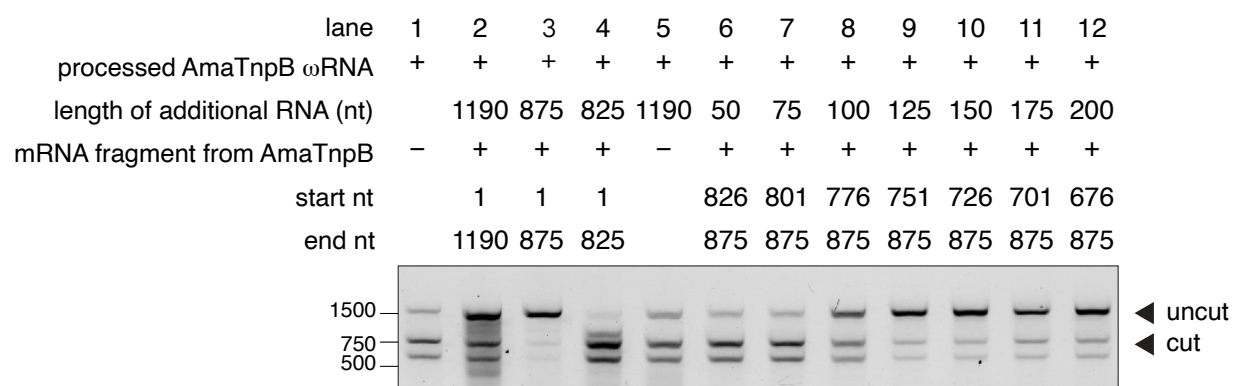

**Figure S1**

Supplement: Supplemental data [file Supp_FigureS1_S5.zip › Supp_FigureS1.pdf]

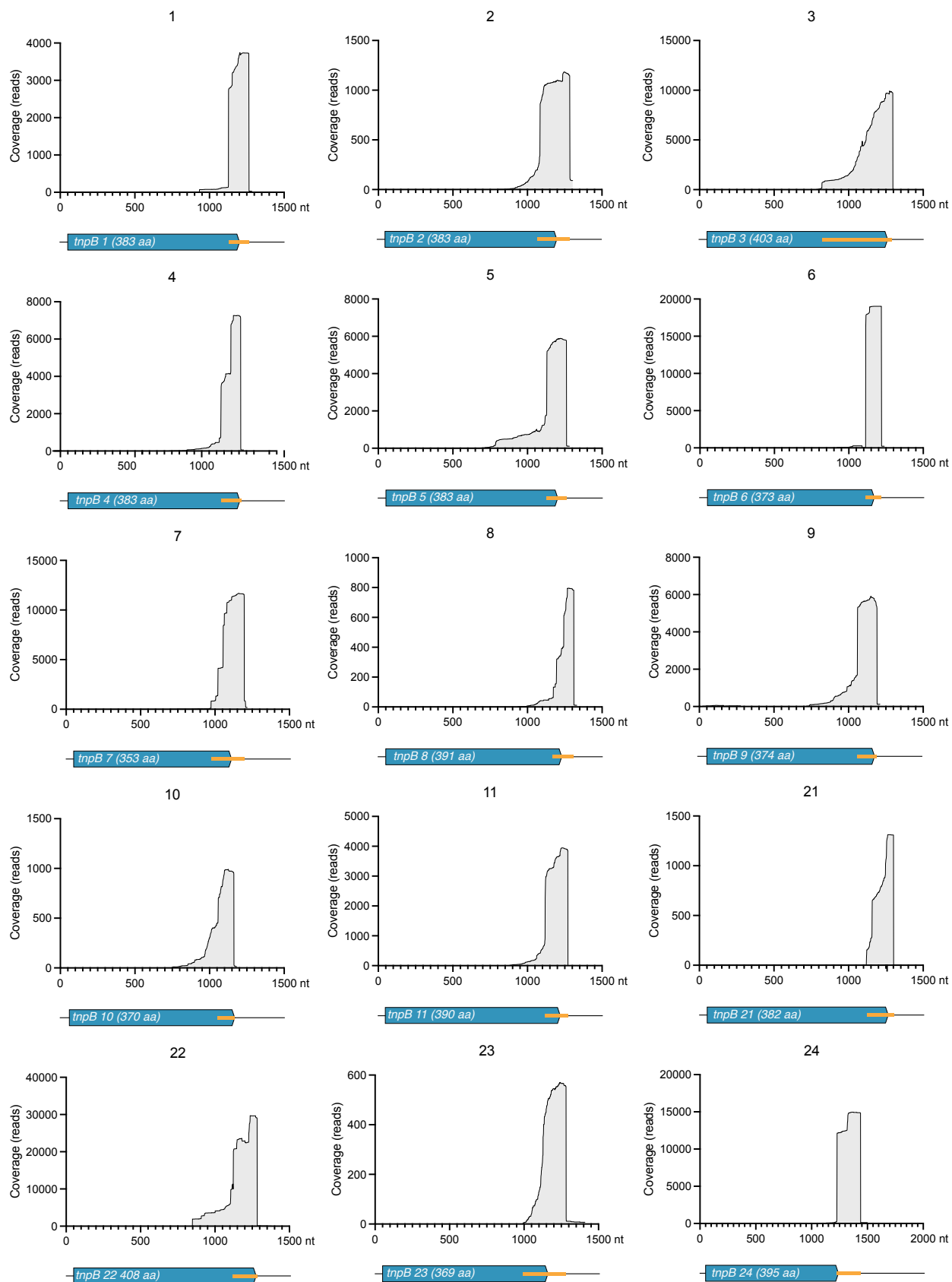

**Figure S2**

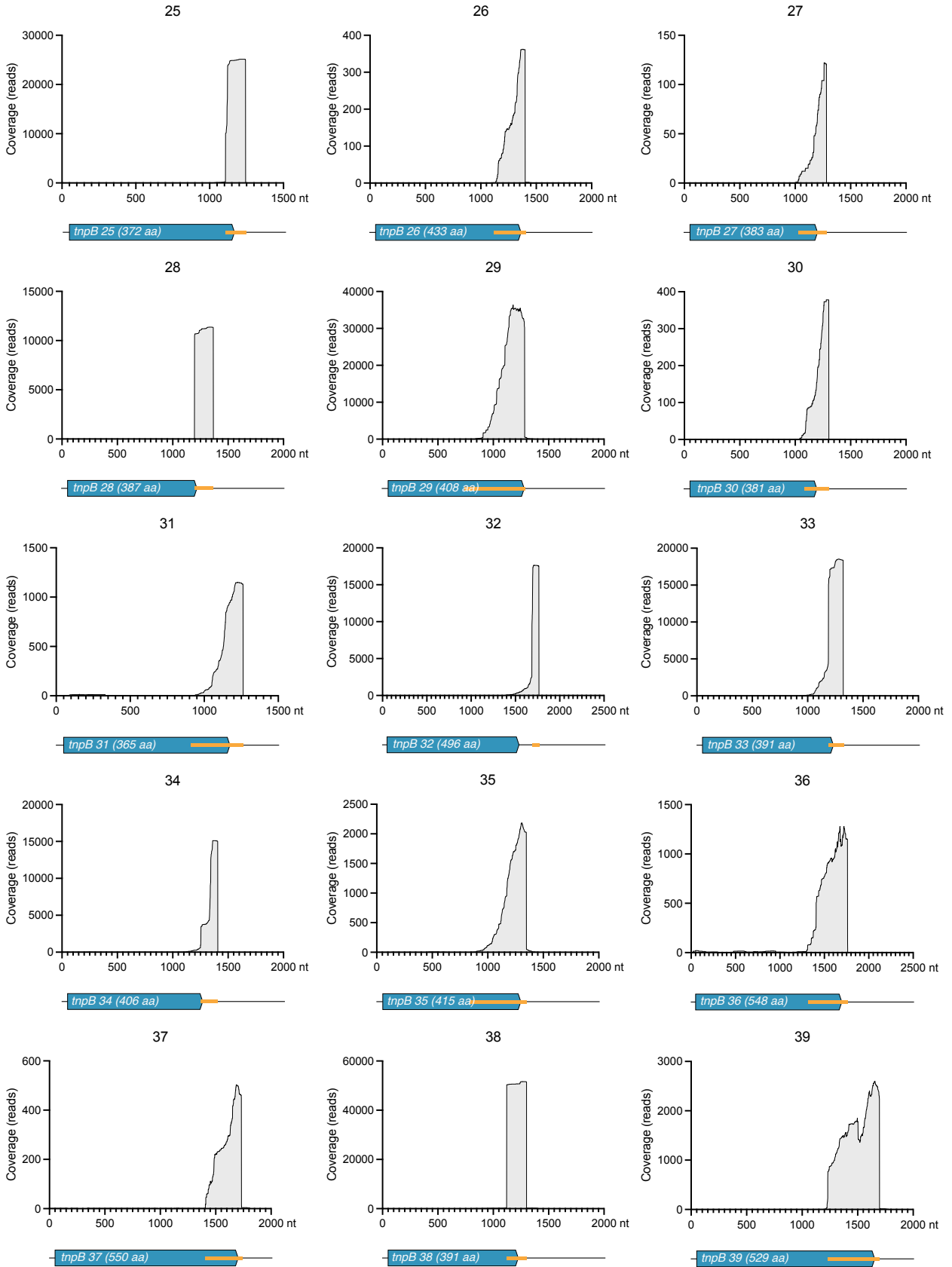

**Figure S2**

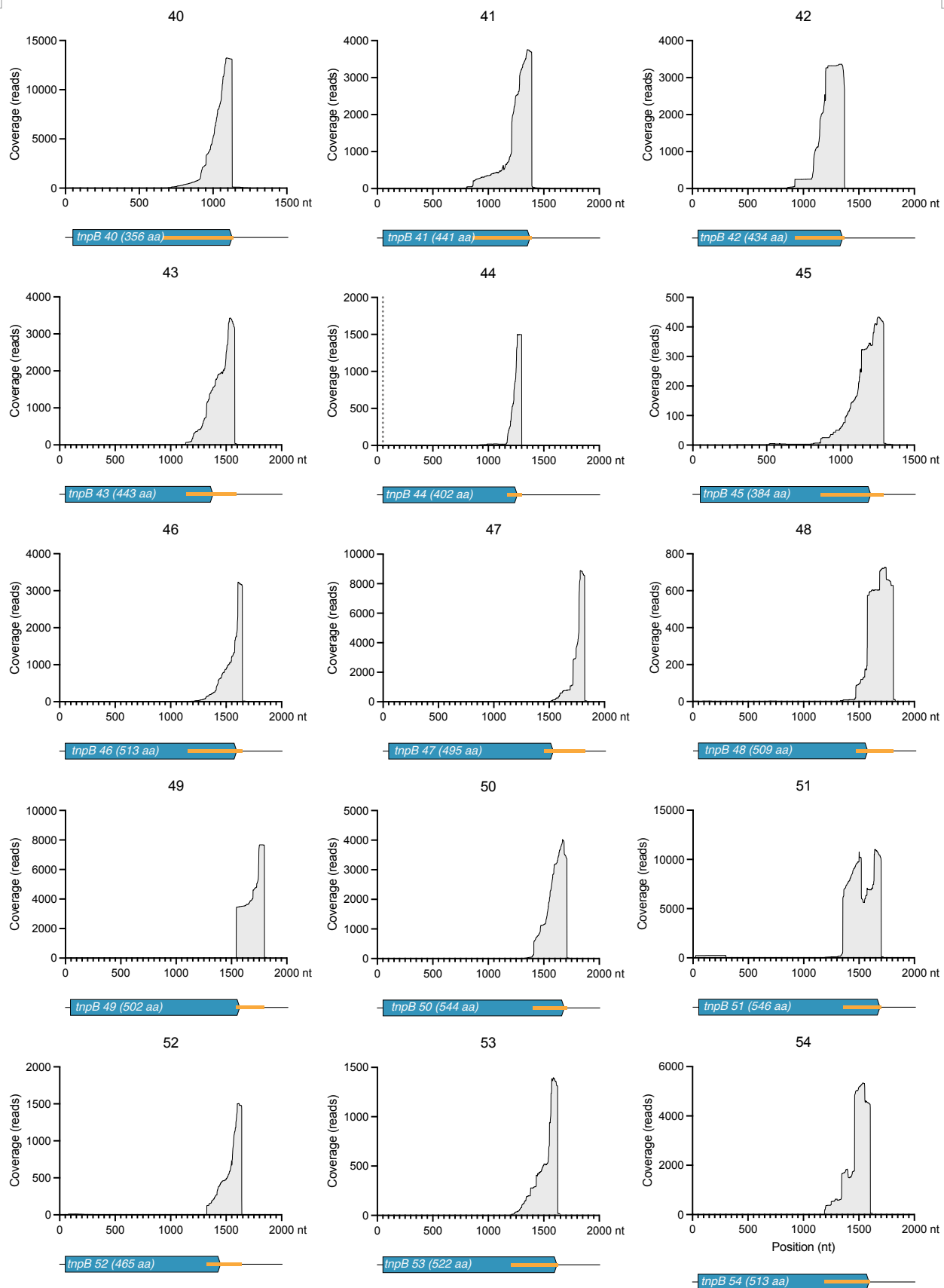

**Figure S2**

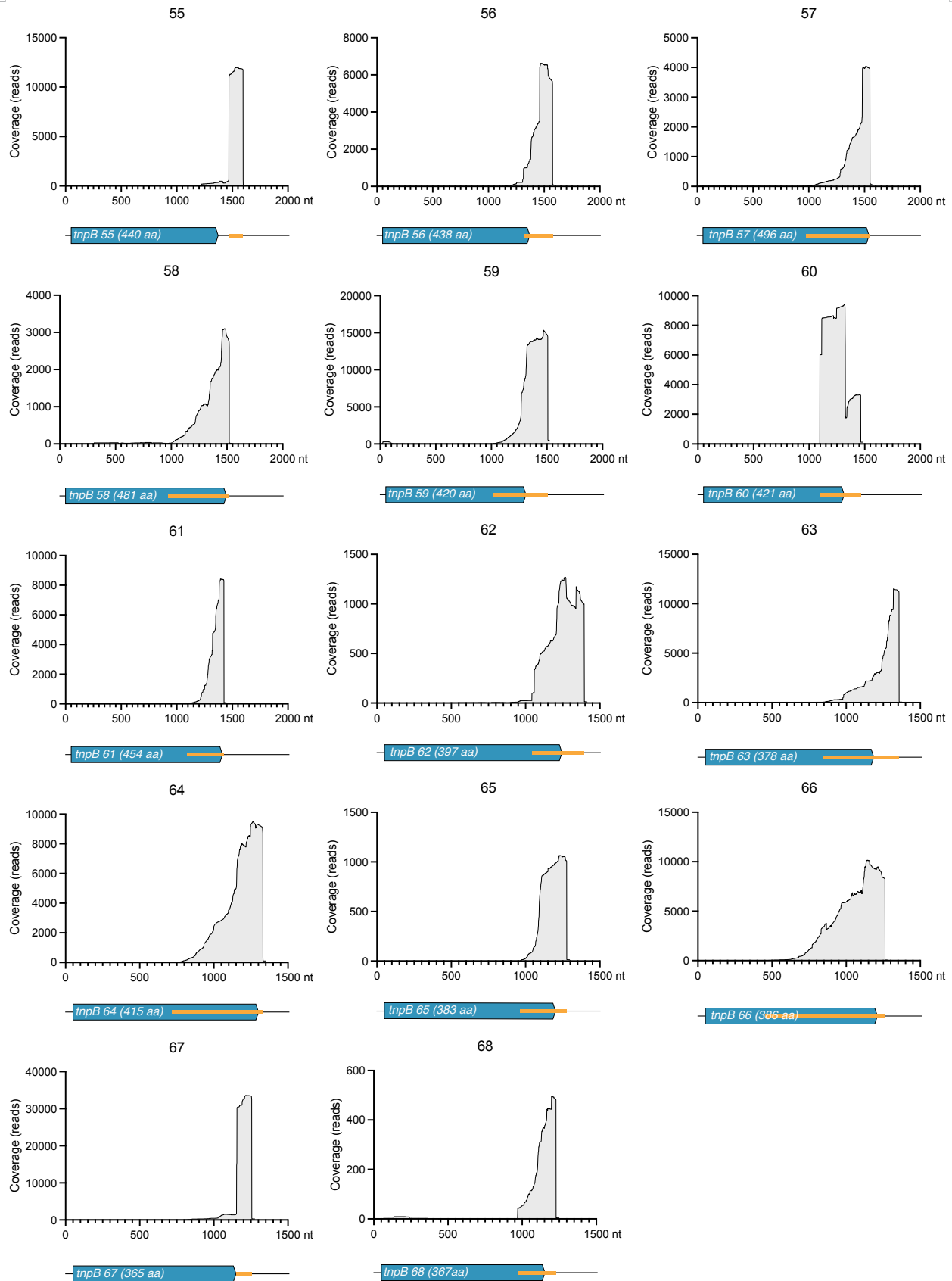

**Figure S2**

Supplement: Supplemental data [file Supp_FigureS1_S5.zip › Supp_FigureS2.pdf]

**A**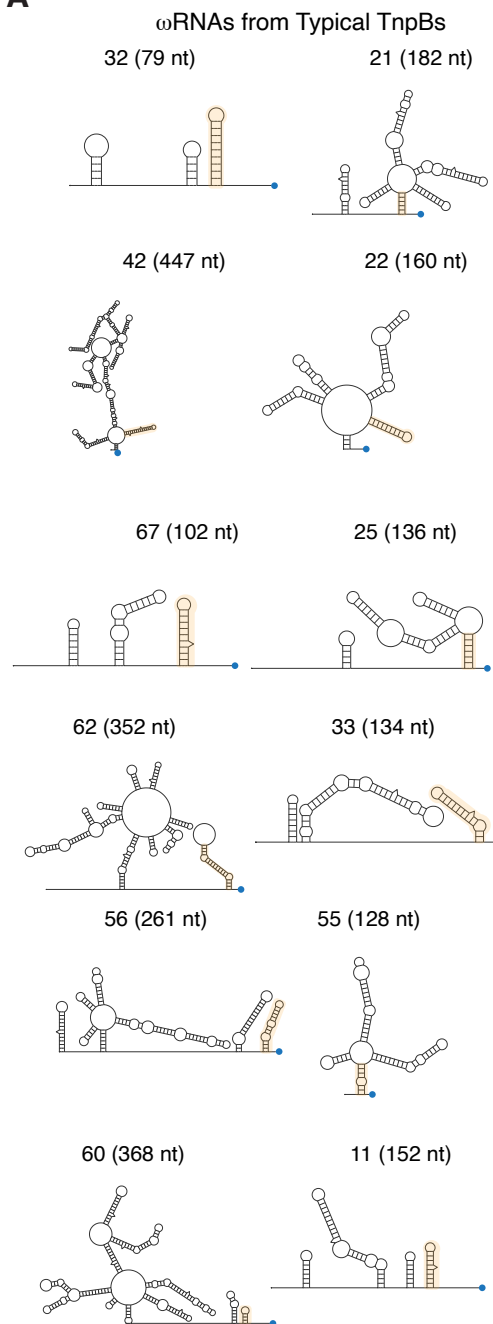**B**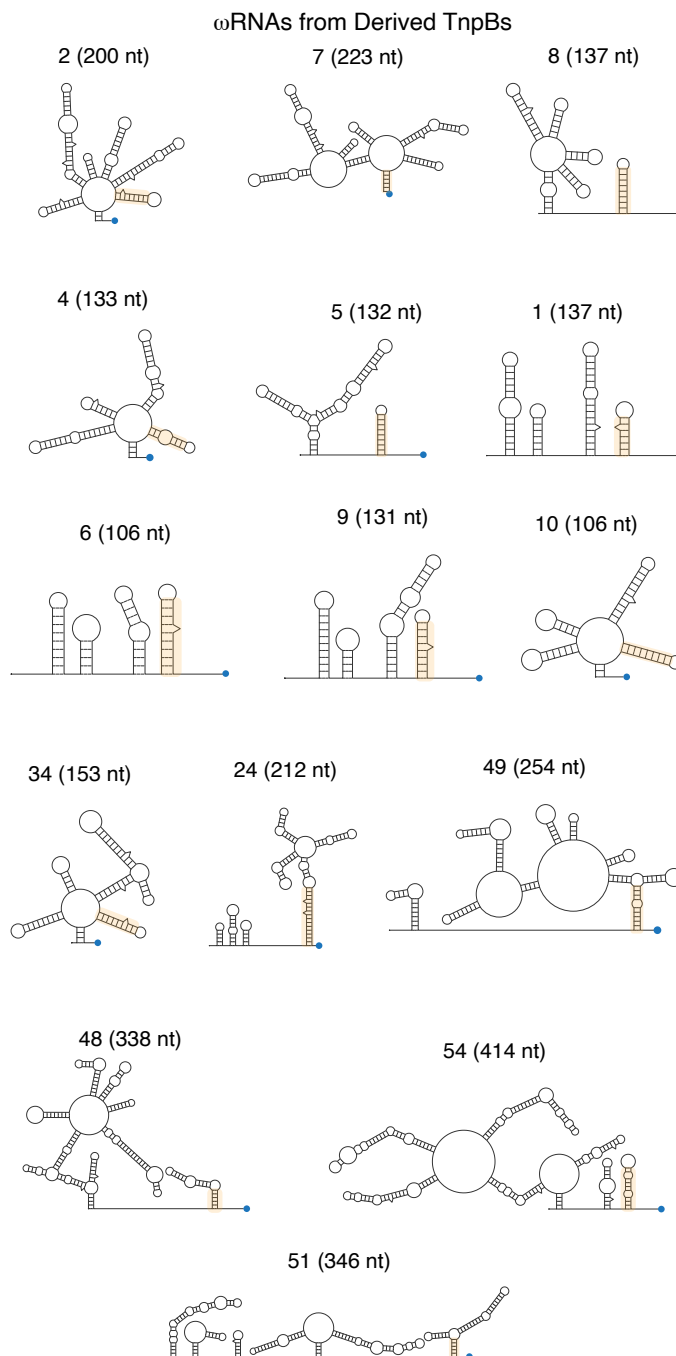**C**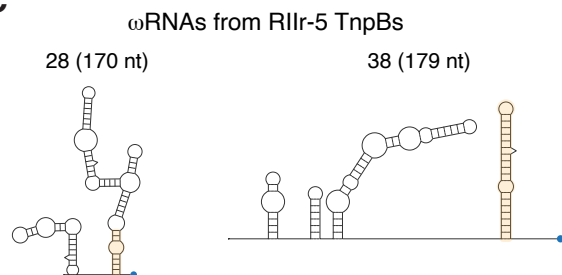**D**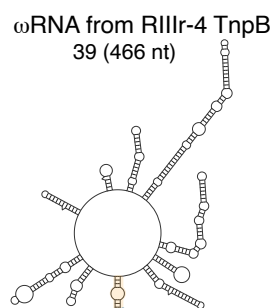**Figure S3**

Supplement: Supplemental data [file Supp_FigureS1_S5.zip › Supp_FigureS3.pdf]

**A**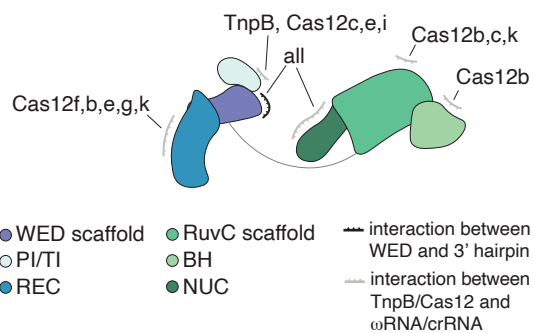**B**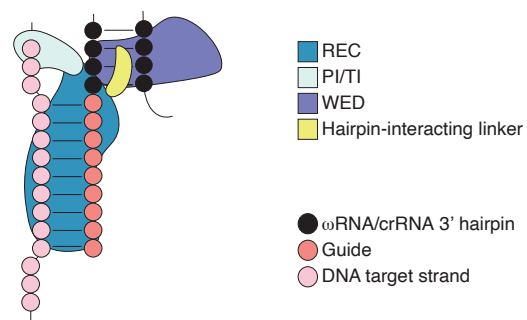**C**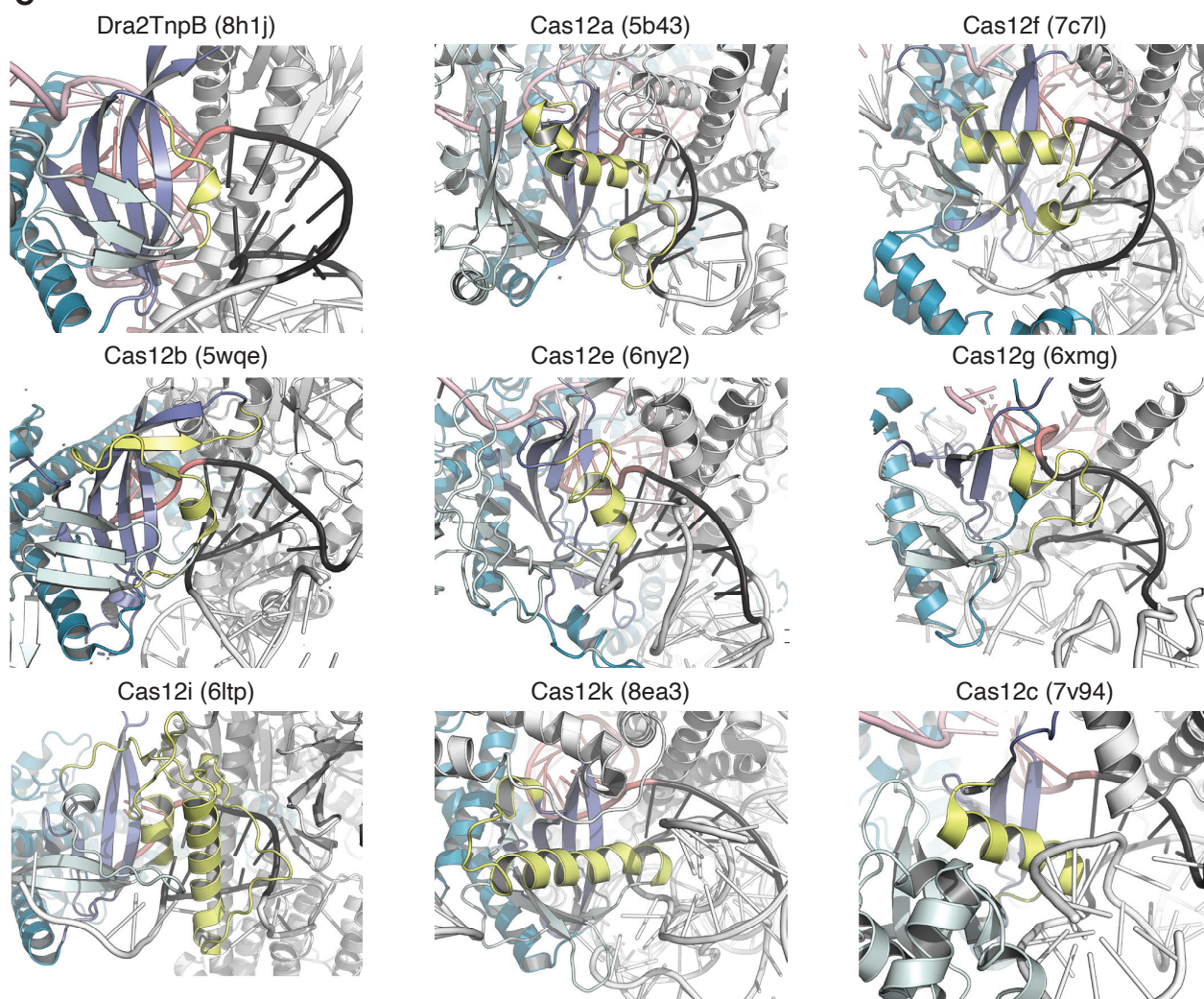**D**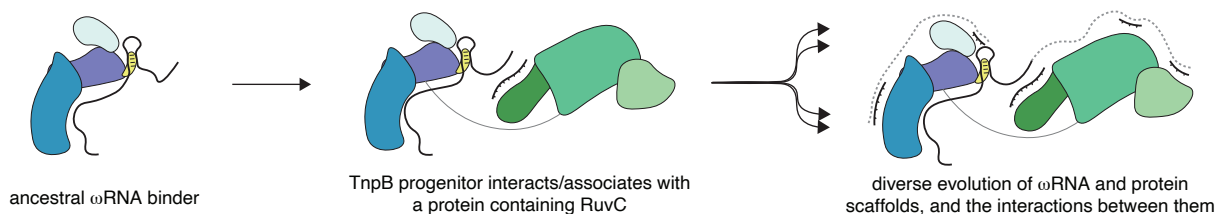**Figure S4**

Supplement: Supplemental data [file Supp_FigureS1_S5.zip › Supp_FigureS4.pdf]

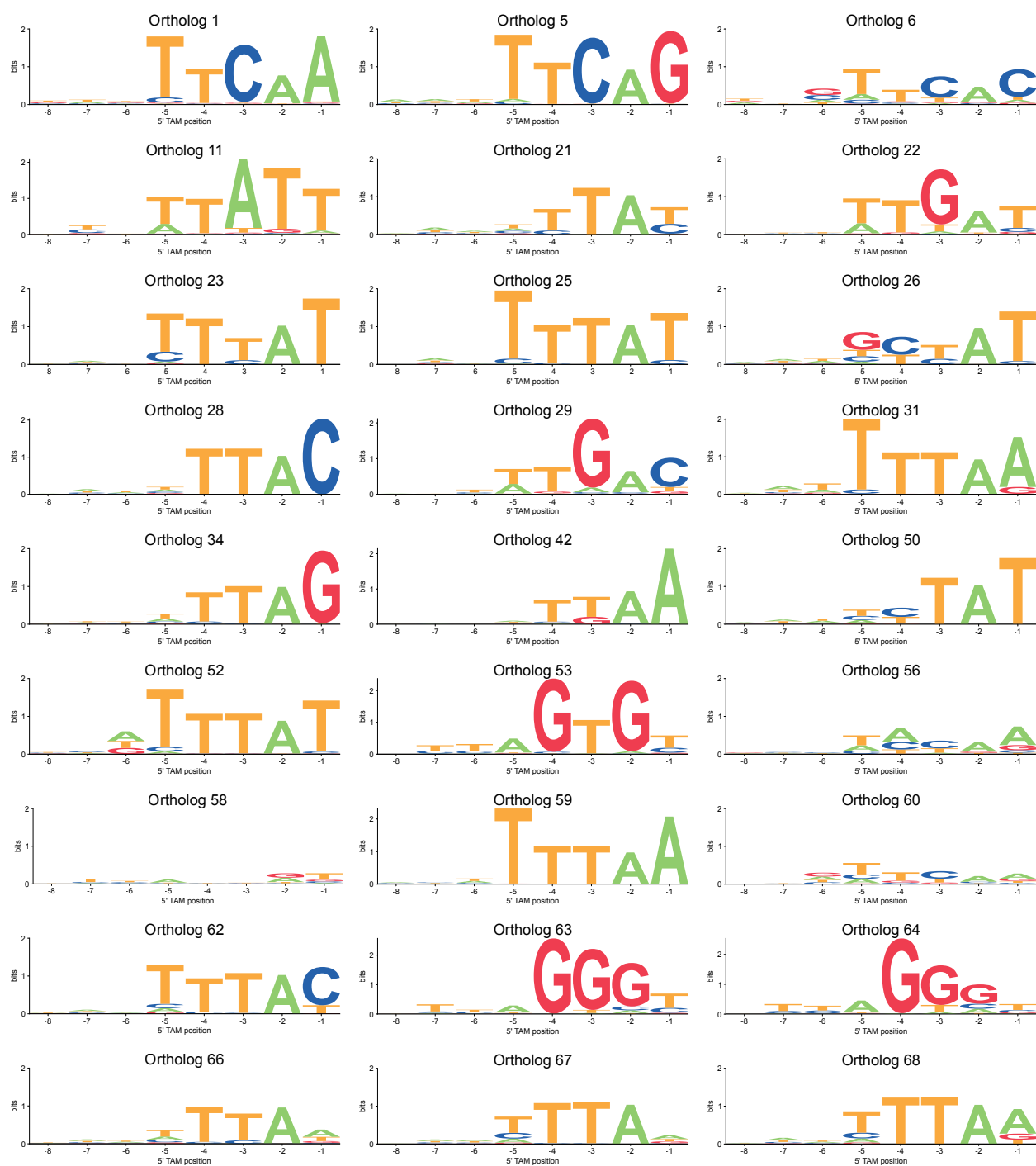

**Figure S5**

Supplement: Supplemental data [file Supp_FigureS1_S5.zip › Supp_FigureS5.pdf]
